# Use of tobacco in schizophrenia: A double‐edged sword

**DOI:** 10.1002/brb3.1433

**Published:** 2019-10-11

**Authors:** Yu Fang, Weidi Wang, Cuizhen Zhu, Guan Ning Lin, Ying Cheng, Junhui Zou, Donghong Cui

**Affiliations:** ^1^ Shanghai Key Laboratory of Psychotic Disorders Shanghai Mental Health Center Shanghai Jiao Tong University School of Medicine Shanghai China; ^2^ Anhui Mental Health Center Anhui China; ^3^ School of Biomedical Engineering Shanghai Jiao Tong University Shanghai China; ^4^ Department of Psychiatry the Seventh People's Hospital of Cixi City Ningbo China

**Keywords:** psychotic symptoms, schizophrenia, self‐medication, tobacco use

## Abstract

**Objective:**

It has been identified that the smoking rate is higher in schizophrenic patients than general population. This study aimed to explore the association between schizophrenia and tobacco use, and provide rational recommendations for clinical care of schizophrenia.

**Methods:**

We recruited 244 patients with schizophrenia and 225 healthy controls. Of schizophrenia patients, 54 patients were untreated with any antipsychotics over the previous 6 months or first‐episode and drug‐naïve. These patients (nonmedication subgroup) were followed up for 8 weeks. The associations between tobacco use and susceptibility to schizophrenia and psychotic symptoms were analyzed.

**Results:**

Although there was no significant difference between schizophrenia patients and healthy controls in the entire sample, stratification analysis showed the rate of smoking was higher in male patients versus healthy controls and that male smokers exhibited higher odds ratios for schizophrenia than nonsmokers. Next, when we repeated analyses in first‐episode patients and healthy controls, significant differences were not observed, indicating tobacco use is an outcome rather than a cause of schizophrenia. Furthermore, among nonmedication subgroup, smokers presented with more severe psychotic symptoms at baseline, and better improvement after medication than nonsmokers, suggesting patients with worse symptoms tend to smoke to relieve symptoms.

**Conclusion:**

This study supports the self‐medication hypothesis. Nonetheless, considering the serious health hazard associated with tobacco use, we should encourage patients to stop smoking. Further investigations are warranted to determine the tobacco constituents that are beneficial or harmful to schizophrenia.

## Graphical abstract

This study investigated the association between tobacco use and susceptibility to schizophrenia and psychotic symptoms. It provides evidence to support the self‐medication hypothesis and provides guidance for clinical care and rehabilitation of schizophrenia.

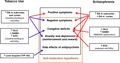

## 1 INTRODUCTION

Schizophrenia is a complex and debilitating psychiatric disorder with a lifetime prevalence of approximately 1% (Agerbo et al., 2015). This mental disorder may shorten life expectancy by 10–20 years (Owen, Sawa, & Mortensen, 2016). Numerous investigations have unveiled high comorbidity between schizophrenia and tobacco use (Chen et al., 2016; Hartz et al., 2018; Parikh, Kutlu, & Gould, 2016). Schizophrenic patients (SPs) exhibit a higher rate of smoking than the general population, even after adjusting for an array of potential confounding factors (Zhang et al., 2013). The use of tobacco is an established cause of physical health problems (e.g., cardiovascular disorders, chronic respiratory disorders, lung cancer, and other malignancies), which may result in early death among individuals with schizophrenia (GBD, 2015 Tobacco Collaborators, 2017; Lucatch, Lowe, Clark, Kozak, & George, 2018). The etiology of predisposition to smoking in schizophrenia remains largely unknown. For the previous three decades, a self‐medication hypothesis provided an interpretation of this observation; the high rate of tobacco use among SPs may be attributed to its effective improvements on cognitive deficit, psychotic and affective symptoms of schizophrenia, and side effects of antipsychotics (Dantas et al., 2017; Kumari & Postma, 2005). However, several recent prospective surveys indicated that tobacco use in adolescents and young adults increased the risk of subsequent schizophrenia in a dose–response pattern. This implies that tobacco use may cause schizophrenia (Scott et al., 2018). In response to these discordances, several researchers have developed alternative theories. One theory proposed that inherent genetic, neurobiological, and environmental factors of schizophrenia predispose patients to smoking, while another theory suggested that schizophrenia and tobacco use share a genetic background (Hu et al., 2018; Lucatch et al., 2018; Šagud et al., 2018).

Meanwhile, several clinical studies reported conflicting evidence, indicating that smokers with schizophrenia exhibited either higher or lower symptom severity than nonsmokers (Aguilar, Gurpegui, Diaz, & De Leon, 2005; Iasevoli, Balletta, Gilardi, Giordano, & de Bartolomeis, 2013; Misiak, Kiejna, & Frydecka, 2015; Zhang et al., 2013). This discrepancy may be the result of differences in the medication status of individuals with schizophrenia (Misiak et al., 2015). Therefore, it is necessary to explore any potential associations in untreated patients. A case–control study involving first‐episode schizophrenia (FES) patients showed that smokers with schizophrenia exhibited more severe positive symptoms than nonsmokers (Zhang et al., 2013), while another study in the same setting reported significantly lower severity of negative symptoms in smoking patients (Misiak et al., 2015). However, the research conducted in this subset of patients remains limited, especially regarding the association between tobacco use and improvement of psychotic symptoms. Further studies are warranted to uncover the significance and reliability of the potential association between tobacco use and psychotic symptoms.

The present study investigated the association between tobacco use and susceptibility to schizophrenia and psychotic symptoms. The aims were to (a) describe the rate of smoking among patients from the Han Chinese population and the association between tobacco use and risk of schizophrenia; (b) determine the relationship between tobacco use and severity of psychotic symptoms prior to the administration of medication; and (c) analyze the effect of tobacco use on the improvement of symptoms following medication.

## 2 PARTICIPANTS AND METHODS

### 2.1 Participants

This study was approved by the Medical Ethics Committee of Shanghai Mental Health Center (SMHC). All participants provided written informed consent in accordance with the tenets of the Declaration of Helsinki.

Schizophrenic patients were recruited from SMHC in China from November 2015 to March 2018. The inclusion criteria for SPs were as follows: diagnosis of schizophrenia according to the International Classification of Diseases, 10th Edition (ICD‐10; World Health Organization, 1993); age 18–65 years; Han Chinese ethnicity. The exclusion criteria were as follows: other concurrent psychiatric disorders defined in the ICD‐10 such as alcohol addiction; ongoing severe physical disorders, pregnancy, or breastfeeding. All patients were assessed via the Mini International Neuropsychiatric Interview (MINI) 6.0.0 (Sheehan & Lecrubier, 2010; Sheehan et al., 1998) to confirm the psychiatric diagnosis. In addition, their medication histories were obtained from medical records. Healthy controls (HCs) were recruited though advertisements at SMHC from December 2010 to October 2016. HCs were matched with SPs in terms of age and gender. At enrollment, healthy volunteers were assessed with MINI 6.0.0 to rule out any psychiatric conditions. Additional exclusion criteria included the following: a history of treatment with psychiatric medications; severe physical disorders.

Following strict screening, 244 SPs (males/females: 124/120; average age: 36.92 ± 13.23 years) and 225 HCs (males/females: 106/119; average age: 36.35 ± 12.48 years) from the Han Chinese population were included in our study. Of the SPs, 54 patients formed the nonmedication subgroup, including 21 first‐episode and drug‐naïve patients and 33 patients untreated with any antipsychotics over the previous 6 months at enrollment. The subgroup was followed up for 8 weeks. As shown in Table 1, SPs exhibited a higher rate of family history than HCs (*χ* ^2^ = 37.79, *p* < .001) and did not differ from HCs with respect to age (*U* = 26,999.50, *p* = .759) and gender (*χ* ^2^ = 0.64, *p* = .422). The range of age at first episode of patients was 10–55 years (25.24 ± 8.79 years), and the range of illness duration was 0–44 years (11.44 ± 11.07 years).

**Table 1:** Demographic characteristics of schizophrenic patients and healthy controls

	HCs (*n* = 225)	SPs (*n* = 244)	Statistics	*p*‐Values
First episode	—	21		
Age at first episode^a^	—	25.24 ± 8.79		
Duration of illness^a^	—	11.44 ± 11.07		
Age^a^	36.35 ± 12.48	36.92 ± 13.23	*U* = 26,999.50	.759
Family history (yes/no)	2/223	43/201	*χ* ^2^ = 37.79	**<.001**
With tobacco use (%)			*χ* ^2^ = 1.23	.268
No	187 (83.1)	193 (79.1)		
Yes	38 (16.9)	51 (20.9)		
Gender (%)			*χ* ^2^ = 0.64	.422
Male	106 (47.1)	124 (50.8)	*χ* ^2^ = 6.03	**.014**
Tobacco use	25 (23.6)	48 (38.7)		
Tobacco naïve	81 (76.4)	76 (61.3)		
Female	119 (52.9)	120 (49.2)	*χ* ^2^ = 6.79	**.009**
Tobacco use	13 (10.9)	3 (2.5)		
Tobacco naïve	106 (89.1)	117 (97.5)		

Abbreviations: HCs, healthy controls; SPs, schizophrenic patients. Bold font indicates statistical significance.

a Mean ± standard deviation.

### 2.2 Clinical assessments

At enrollment, SPs and HCs were both assessed with MINI 6.0.0 to define the diagnosis of psychiatric disorders. In addition, the Positive and Negative Syndrome Scale (PANSS; Si et al., 2004) and the Brief Psychiatric Rating Scale (BPRS; Zhang, 1984) were used to evaluate the psychotic symptoms of SPs. Trained research assessors rated every item of the PANSS and BPRS using a 7‐point scale (1 = absent to 7 = extreme) at routine visits (enrollment and follow‐up at 4 and 8 weeks). The self‐report questionnaire was used to collect information concerning gender, age, age at first episode, duration of illness, family history, and tobacco use.

### 2.3 Statistical analysis

The normal distribution of continuous variables was tested with the one‐sample Shapiro–Wilk test. Student's *t* test, Mann–Whitney *U* test, or chi‐square test was used to compare demographic characteristics between SPs and HCs. These sociodemographic variables (i.e., age, gender, and family history) were modeled as confounders. A logistic regression, with tobacco use as an independent variable, was used to calculate crude and adjusted odds ratios (OR) for the diagnosis of schizophrenia. Given the difference observed between the genders in terms of tobacco use, a gender‐stratified logistic regression was performed. In addition, a logistic regression for FES patients was performed to determine tobacco use as the cause or outcome of schizophrenia.

Furthermore, the association between tobacco use and psychotic symptoms was analyzed in the nonmedication subgroup. The subgroup consisted of 32 patients treated with single antipsychotics, 14 with one antipsychotics and adjuvant drugs, and eight with combined antipsychotics. The doses of antipsychotics were converted to equivalent doses of olanzapine based on the defined daily doses (Leucht, Samara, Heres, & Davis, 2016) and were compared between smokers and nonsmokers. Student's *t* test or Mann–Whitney *U* test was used to compare the PANSS and BPRS scores at baseline between smokers and nonsmokers. Subsequently, a repeated measures analysis of variance (RM‐ANOVA) was performed. Additionally, two‐way RM‐ANOVA on the PANSS and BPRS scores was performed, with time and tobacco use as within and between factors, and drug dosage as covariate. The Bonferroni post hoc test was conducted, as appropriate.

All analyses were conducted using the SPSS version 22.0 (IBM Corp.). Figures were drawn using GraphPad Prism 6.0 (GraphPad Software). A *p* < .05 denoted statistical significance.

## 3 RESULTS

### 3.1 Tobacco use might be an outcome rather than a cause of schizophrenia

Overall, the rates of smoking were 20.9% and 16.9% in SPs and HCs, respectively; however, the difference was not statistically significant (*χ* ^2^ = 1.23, *p* = .268; Table 1). Notably, a stratification analysis according to gender found that among male participants, the rates of smoking were 38.7% and 23.6% in SPs and HCs, respectively; this difference was statistically significant (*χ* ^2^ = 6.03, *p* = .014). Among female participants, these rates were 2.5% and 10.9%, respectively, and the difference was statistically significant (*χ* ^2^ = 6.79, *p* = .009; Table 1).

In the entire study sample, logistic regression showed that there was no significant difference in the odds of having schizophrenia between tobacco users and tobacco naïve subjects (OR_crude_ = 1.30, 95% confidence interval [CI] = 0.82–2.07, *p* = .269; OR_adjusted_ = 1.32, 95% CI = 0.79–2.22, *p* = .293). Furthermore, gender‐stratified logistic regression found that tobacco users showed an increased odds of schizophrenia (OR_crude_ = 2.05, 95% CI = 1.15–3.64, *p* = .015) in males and a lower odds (OR_crude_ = 0.21, 95% CI = 0.06–0.75, *p* = .017) in females when compared with tobacco naïve subjects (Table 2). The statistical significances were maintained even after adjusting for confounders in both males (OR_adjusted_ = 2.37, 95% CI = 1.28–4.38, *p* = .006) and females (OR_adjusted_ = 0.17, 95% CI = 0.04–0.72, *p* = .016; Table 2).

**Table 2:** Odds ratios of tobacco use for the diagnosis of schizophrenia

	Odds ratios					
	Crude			Adjusted for confounders		
	Estimate	95% CI	*p* Value	Estimate	95% CI	*p*‐Values
All schizophrenic patients included						
Both	1.30	0.82, 2.07	.269	1.32	0.79–2.22	.293
Male	2.05	1.15, 3.64	**.015**	2.37	1.28–4.38	**.006**
Female	0.21	0.06, 0.75	**.017**	0.17	0.04–0.72	**.016**
FES patients included						
Both	1.16	0.37, 3.63	.802	1.41	0.39–5.18	.602
Male	1.22	0.30, 4.93	.785	1.29	0.30–5.66	.733
Female	0.91	0.11, 7.74	.928	0.80	0.03–24.27	.899

Abbreviations: CI, confidence interval; FES, first‐episode schizophrenia. Bold font indicates statistical significance.

We repeated the analysis in FES patients and HCs to determine whether tobacco use was the cause or outcome of schizophrenia. The rates of smoking were 19.0% and 16.9% in FES patients and HCs, respectively. The difference was not statistically significant (*p* = .765, Fisher's exact test). Among the male participants, the rates of smoking were 27.3% and 23.6% in FES patients and HCs, respectively. Among the female participants, these rates were 10.0% and 10.9%, respectively. The aforementioned statistical significance in the case–control comparisons disappeared for both males (*p* = .517, Fisher's exact test) and females (*p* = .703, Fisher's exact test). Furthermore, the logistic regression on schizophrenia risk indicated that there were no significant difference between tobacco users and tobacco naïve subjects in the entire sample (OR_crude_ = 1.16, 95% CI = 0.37–3.63, *p* = .802; OR_adjusted_ = 1.41, 95% CI = 0.39–5.18, *p* = .602), in males (OR_crude_ = 1.22, 95% CI = 0.30–4.93, *p* = .785; OR_adjusted_ = 1.29, 95% CI = 0.30–5.66, *p* = .733), and females (OR_crude_ = 0.91, 95% CI = 0.11–7.74, *p* = .928; OR_adjusted_ = 0.80, 95% CI = 0.03–24.27, *p* = .899; Table 2). In summary, these findings indicated that tobacco use might be an outcome rather than a cause of schizophrenia.

### 3.2 More severe psychotic symptoms observed in smokers

**Figure 1:**
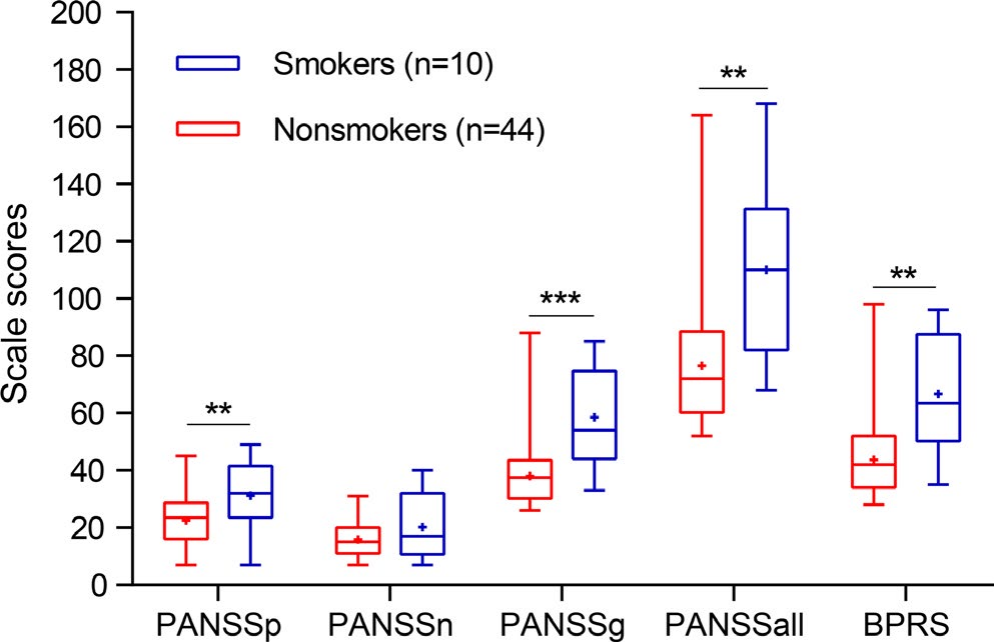
The association between tobacco use and psychotic symptoms prior to the administration of medication. Smokers exhibited higher PANSS positive scores (PANSSp), PANSS general psychopathology scores (PANSSg), PANSS total scores (PANSSall), and BPRS scores compared with nonsmokers. The PANSS negative scores (PANSSn) did not differ between smokers and nonsmokers. “+” represented mean. The bar represented min and max scores, and the box represented upper quartile, median, and lower quartile. *p < .05, **p < .01, ***p < .001

The nonmedication subgroup (54 patients) was analyzed to assess the association between tobacco use and psychotic symptoms. At baseline, smokers exhibited higher PANSS positive scores (*t* = −2.70, *p* = .009), PANSS general psychopathology scores (*U* = 59.50, *p* < .001), PANSS total scores (*U* = 70.00, *p* = .001), and BPRS scores (*U* = 74.50, *p* = .001) versus nonsmokers (Table 3, Figure 1). However, the PANSS negative scores did not differ between smokers and nonsmokers (*t* = −1.15, *p* = .275; Table 3, Figure 1). These findings implied that smokers suffered from more severe positive symptoms and general psychopathology symptoms.

**Table 3:** The association between tobacco use and psychotic symptoms before medication

Scale scores (mean ± *SD*)	Tobacco use		Statistics	*p* Value
	No (*n* = 44)	Yes (*n* = 10)		
PANSS positive scores	22.52 ± 8.50	31.30 ± 12.35	*t* = −2.70	**.009**
PANSS negative scores	15.93 ± 6.22	20.20 ± 11.31	*t* = −1.15	.275
PANSS general psychopathology scores	38.16 ± 10.59	58.50 ± 17.25	*U* = 59.50	**<.001**
PANSS total scores	76.61 ± 21.01	110.00 ± 31.05	*U* = 70.00	**.001**
BPRS scores	43.77 ± 12.84	66.80 ± 22.46	*U* = 74.50	**.001**

Abbreviations: PANSS, Positive and Negative Syndrome Scale; *SD*, standard deviation. Bold font indicates statistical significance.

### 3.3 Improvement of the therapeutic efficacy of antipsychotics through tobacco use

**Figure 2:**
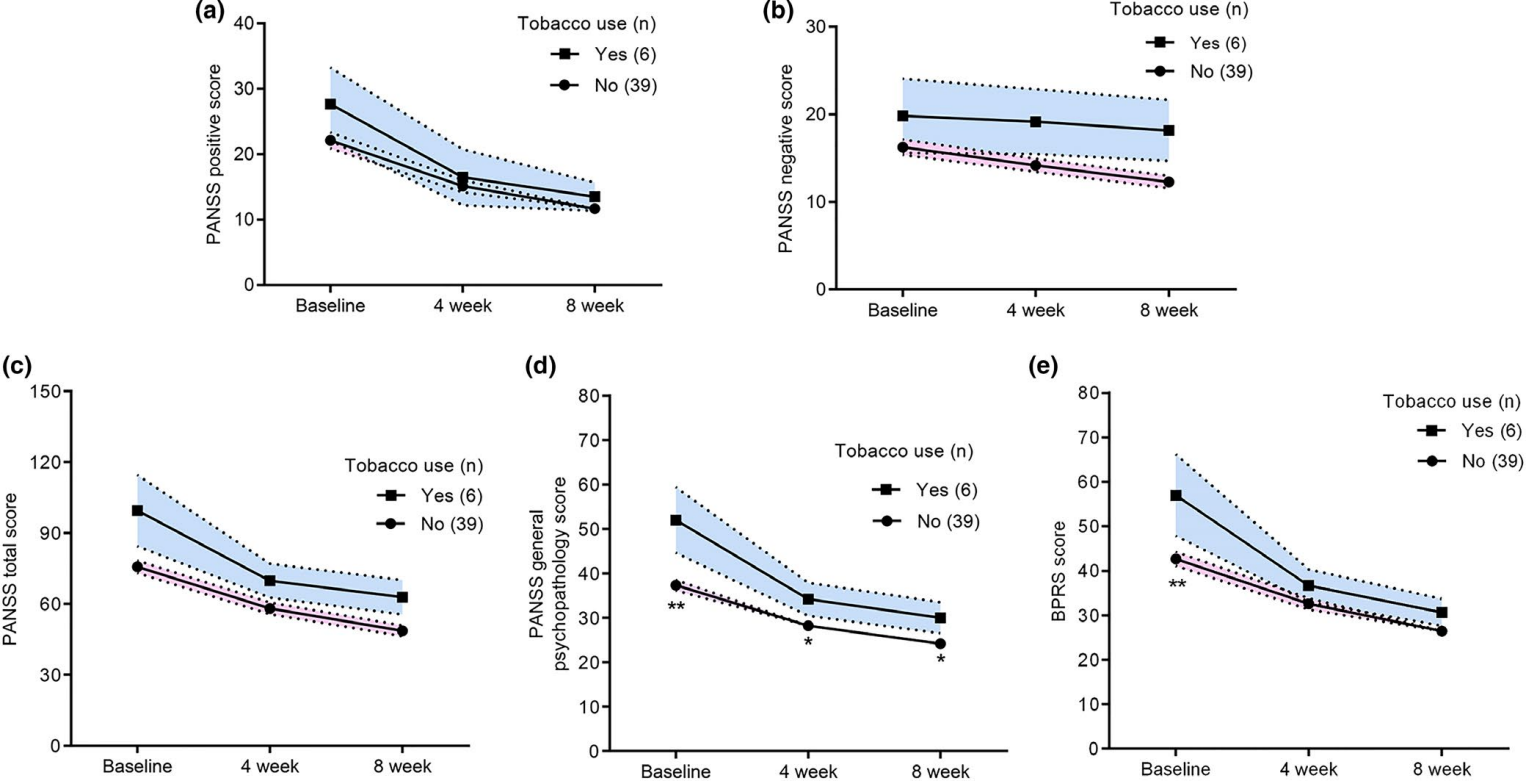
The association between tobacco use and improvement of psychotic symptoms. (a, b, c) There were no significant interactions between tobacco use and time on PANSS positive scores, PANSS negative scores, and PANSS total scores. (d, e) There were significant interactions between tobacco use and time on PANSS general psychopathology scores and BPRS scores. The bars represented mean ± standard error. *p < .05, **p < .01 compared with smoker, Bonferroni post hoc test following two‐way RM‐ANOVA

The 54 patients in the nonmedication subgroup were followed up for 8 weeks. Of those, nine patients were lost to follow‐up, and the remaining 45 patients were included for further analysis. We observed a trend toward prescription of higher doses of olanzapine equivalents in smokers versus nonsmokers, though the difference was not statistically significant (10.10 ± 4.61 vs. 8.83 ± 4.93 mg, respectively, *t* = −0.74, *p* = .466). RM‐ANOVA revealed the great recovery of patients in the PANSS positive score (*F* = 66.34, *p* < .001), PANSS negative score (*F* = 17.77, *p* < .001), PANSS general psychopathology score (*F* = 103.40, *p* < .001), PANSS total score (*F* = 109.03, *p* < .001), and BPRS score (*F* = 83.54, *p* < .001) following medication (Table 4). An additional two‐way RM‐ANOVA showed that the interaction between tobacco use and time exerted notable effects on the PANSS general psychopathology score (*F* = 4.53, *p* = .017), and the BPRS score (*F* = 3.51, *p* = .040; Table 4, Figure 2). However, we did not observe any significant interaction between tobacco use and time on PANSS positive score, PANSS negative score, and PANSS total score (all *p* > .05; Table 4, Figure 2). These results indicated tobacco use might improve the therapeutic efficacy of antipsychotics on general psychopathology symptoms.

**Table 4:** Change in the PANSS and BPRS scores following medication

Scale scores (Mean ± *SD*)	Sample (*n*)	Baseline	4 week	8 week	Statistics	*p*‐Values
PANSSp	All (45)	22.87 ± 8.67	15.31 ± 6.47	11.93 ± 4.59	*F* = 66.34	**<.001** a
	Smokers (6)	27.67 ± 13.66	16.50 ± 10.46	13.50 ± 5.32	*F* = 0.95	.397^b^
	Nonsmokers (39)	22.20 ± 7.68	15.20 ± 5.85	11.66 ± 4.67		
PANSSn	All (45)	16.73 ± 6.39	14.84 ± 5.57	13.07 ± 6.39	*F* = 17.77	**<.001** a
	Smokers (6)	19.83 ± 10.36	19.17 ± 9.07	18.17 ± 8.52	*F* = 0.39	.682^b^
	Nonsmokers (39)	16.40 ± 5.87	14.43 ± 4.85	12.57 ± 4.51		
PANSSg	All (45)	39.29 ± 10.49	29.00 ± 6.84	24.93 ± 6.08	*F* = 103.40	**<.001** a
	Smokers (6)	52.00 ± 18.04	34.17 ± 9.11	30.00 ± 8.58	*F* = 4.53	**.017** b
	Nonsmokers (39)	37.66 ± 7.70	28.63 ± 6.31	24.60 ± 5.42		
PANSSall	All (45)	78.89 ± 21.22	59.67 ± 16.12	50.53 ± 15.25	*F* = 109.03	**<.001** a
	Smokers (6)	99.50 ± 36.82	69.83 ± 17.59	62.83 ± 17.83	*F* = 2.94	.065^b^
	Nonsmokers (39)	76.26 ± 16.74	58.91 ± 15.89	49.40 ± 14.62		
BPRS score	All (45)	44.60 ± 12.83	33.13 ± 8.12	27.02 ± 6.87	*F* = 83.54	**<.001** a
	Smokers (6)	57.00 ± 22.44	36.67 ± 9.00	30.67 ± 7.42	*F* = 3.51	**.040** b
	Nonsmokers (39)	42.83 ± 10.05	32.97 ± 8.24	26.89 ± 6.94		

Abbreviations: PANSS, Positive and Negative Syndrome Scale; PANSSall, PANSS total scores; PANSSg, PANSS general psychopathology scores; PANSSn, PANSS negative scores; PANSSp, PANSS positive scores; *SD*, standard deviation. Bold text indicates statistical significance.

a RM‐ANOVA.

b Two‐way RM‐ANOVA adjusting for drug dosage, *p*‐values of interactions between tobacco use and time.

## 4 DISCUSSION

In the present study, among all participants, the rate of smoking was not significantly different between SPs and HCs. However, among male participants, the rate of smoking in SPs was higher than that observed in HCs. Among female participants, the rate of smoking was higher in HCs. Notably, the statistically significant differences were not maintained, following the repeated analysis in FES patients and HCs. Furthermore, prior to the administration of medications, smokers presented with more severe positive and general psychopathology symptoms. Following medication, tobacco use exerted a beneficial effect on the symptoms.

**Figure 3:**
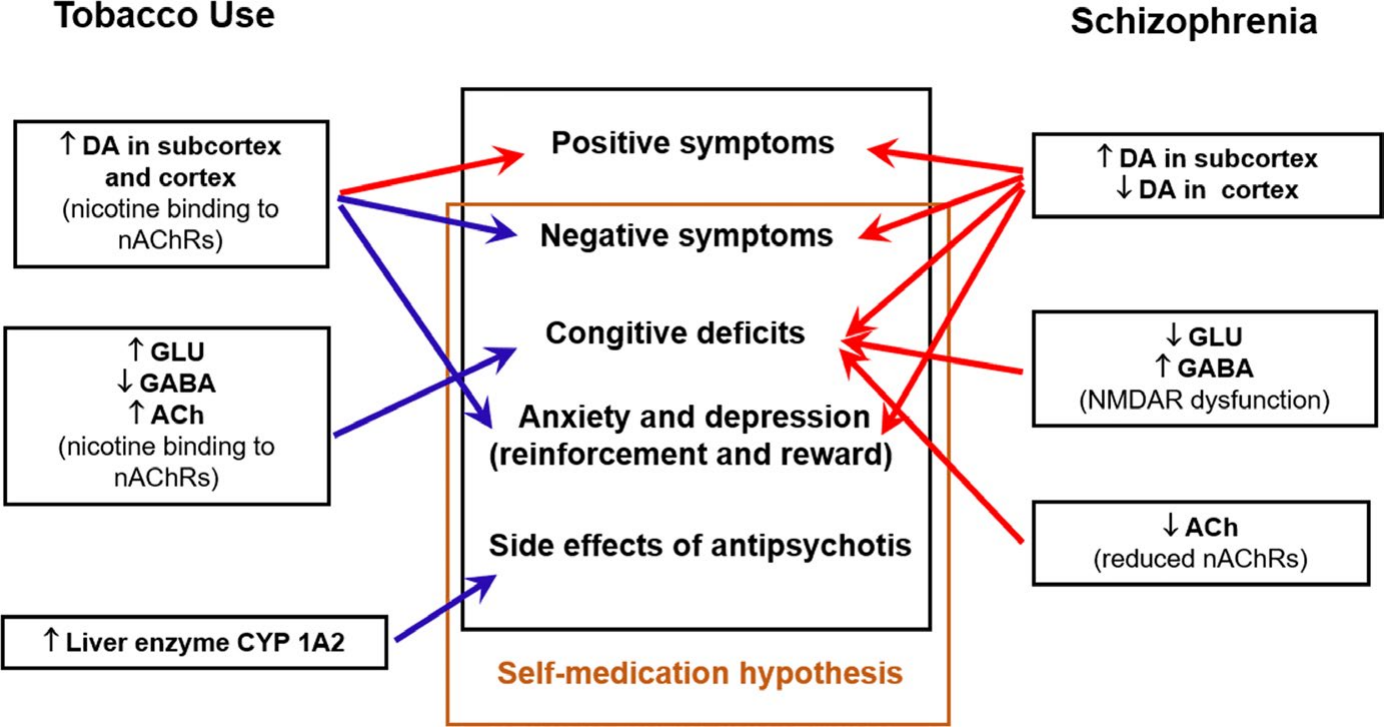
The potential mechanisms of the self‐medication hypothesis. Neurotransmitter dysfunction is the important pathological mechanism of schizophrenia. Nicotine binds to the nicotinic acetylcholine receptors, which are expressed in the human brain and influence neurotransmitter systems, thereby normalizing neurotransmitter dysfunction and improving disease. Red arrows indicate detrimental effects, whereas blue arrows indicate beneficial effects. Ach, acetyl choline; DA, dopamine; GABA, gamma‐aminobutyric acid; GLU, glutamic acid; nAChRs, nicotine acetylcholine receptors

Patients with schizophrenia exhibit a higher rate of tobacco use and a lower rate of smoking cessation versus the general population (De Leon & Diaz, 2005; Li et al., 2019). Two meta‐analyses found that the rate of smoking in Chinese male and female SPs was 59.1% and 4.3%, respectively (Cao et al., 2015; Li et al., 2016). In addition, a prospective and multicenter survey in China reported that the rate of smoking was 13.9% in the whole study sample (26.2% in males and 3.5% in females; Wang et al., 2010). The smoking rate observed in the present study corroborated this finding. Although there was no difference in the overall sample comparison, our gender stratification analysis showed that the rate of smoking was higher in male SPs versus HCs, and opposite in females. Intriguingly, a birth cohort study conducted in Finland found that heavy smoking increased the risk of psychosis, whereas light smoking seemed to exert a protective effect (Mustonen et al., 2018). We speculate that the gender differences reported in our study may be attributed to social and cultural factors. In China, female smokers are markedly fewer and exhibit lighter smoking habits versus males (Wang et al., 2010; Zhang et al., 2013). Besides, the small number of smoking females indicated these findings about females should be interpreted with caution. Moreover, our results remained statistically significant even after adjusting for family history. If the association between tobacco use and schizophrenia was the result of genetic confounding, a steeper decline would be expected. Coincidentally, a co‐relative analysis found a higher risk of nonaffective psychosis among heavy smokers versus their nonsmoking twin (Mustonen et al., 2018). Therefore, the association between tobacco and schizophrenia is unlikely to be completely explained by genetic factors. However, when we repeated the analysis in FES patients and HCs, the statistically significant differences observed in the case–control comparisons were not maintained. This suggests that tobacco use is an outcome rather than a cause of schizophrenia. The self‐medication hypothesis may provide an interpretation, which states that patients with schizophrenia smoke to alleviate clinical symptoms, cognitive deficits, and the side effects of antipsychotic medications (Lucatch et al., 2018). The potential neurobiological mechanism involved in this process is shown in Figure 3. An established hypothesis proposed that neurotransmitter dysfunction (e.g., dopamine, glutamate, gamma‐aminobutyric acid, and acetylcholine) is the important pathological mechanism of schizophrenia (Devor et al., 2017; Dineley, Pandya, & Yakel, 2015; McCutcheon, Abi‐Dargham, & Howes, 2019; Wierońska & Pilc, 2019). Nicotine—the addictive ingredient in tobacco—binds to the nicotinic acetylcholine receptors, which are expressed in the human brain and influence neurotransmitter systems, thereby normalizing neurotransmitter dysfunction and improving disease (Lucatch et al., 2018).

In order to test the hypothesis, we analyzed psychotic symptoms in nonmedication subgroup. We observed that smokers suffered from more severe positive and general psychopathology symptoms than nonsmokers, but there was no difference in negative symptoms between the two. We suspect there are two potential reasons for this phenomenon. The first is that tobacco use may aggravate psychotic symptoms, especially positive symptoms. A study involving Chinese FES patients reported similar results to those obtained in the present study (Zhang et al., 2013). We assume that the potential mechanism is an elevated release and utilization of dopamine in the mesolimbic dopaminergic system by nicotine, which has been strongly associated with positive symptoms (Brody et al., 2006; Kesby, Eyles, McGrath, & Scott, 2018; Li et al., 2004; Montgomery, Lingford‐hughes, Egerton, Nutt, & Grasby, 2007). The other potential reason is self‐medication hypothesis. It suggests patients with worse condition tend to smoke to relieve symptoms. Several studies, which yielded lower severity of negative symptoms in heavy smokers versus nonsmokers, support this opinion (Misiak et al., 2015; Ziedonis, Kosten, Glazer, & Frances, 1994). Of note, our results showed better improvement of general psychopathology symptoms (cognitive and affective symptoms) after medication in smokers versus nonsmokers (Mohr et al., 2004). Another study reported that clozapine in smokers was more effective (Mcevoy, Freudenreich, & Wilson, 1999). The findings of these two studies are also concordant with this hypothesis. In addition to the above neurobiological mechanisms, tobacco use increases the activity of the metabolic enzyme cytochrome P450 1A2. This effect accelerates the decomposition of antipsychotics (especially clozapine and olanzapine) in the body and reduces drug concentration in the blood, resulting in fewer side effects and improved adherence to the medication regimen (Šagud et al., 2009). Because of that, as shown in the present and previous studies, patients who smoke are prescribed higher doses of antipsychotics.

Collectively, the present study supports the self‐medication hypothesis. However, a review concluded that tobacco is less beneficial on psychotic symptoms than generally assumed (Manzella, Maloney, & Taylor, 2015). Moreover, the harmful effects of tobacco use on physical health (i.e., increased risk of cardiovascular diseases and lung cancer) that account for early mortality among patients with schizophrenia certainly outweigh any possible benefits (Hahn et al., 2013; Manzella et al., 2015). Because of the complex composition of tobacco, it is important to determine the ingredients that are beneficial or harmful to psychotic symptoms.

Several limitations should be considered when interpreting these results. Firstly, the small number of patients in the nonmedication group indicates that these findings should be generalized with caution. Secondly, we did not obtain data regarding other smoking variables (e.g., the number of cigarettes per day, age of smoking onset, and levels of nicotine dependence), which should be considered in the future. Despite of these limitations, this study provides guidance for clinical care and rehabilitation of schizophrenia to some extent. Further investigations regarding the pros and cons of tobacco use in the setting of schizophrenia are warranted.

## CONFLICT OF INTEREST

None declared.

## Data Availability

The data that support the findings of this study are available from the corresponding author upon reasonable request.
